# Profil sociodémographique et clinique d’une population de consultants dans un service de psychiatrie d’un hôpital général au Sud tunisien

**DOI:** 10.11604/pamj.2023.44.169.28846

**Published:** 2023-04-12

**Authors:** Yosra Mejdoub, Maissa Ben Jmaa, Wafa Abbes, Mouna Abbes, Sourour Yaich, Khaoula Mdhaffar, Jamel Damak, Houssem Hassen, Jihene Trablesi, Latifa Ghanmi

**Affiliations:** 1 Service de Médecine Communautaire et d´Epidémiologie, CHU Hédi Chaker, Sfax, Tunisie,; 2 Service de Psychiatrie, Hôpital Régional de Gabès, Gabès, Tunisie

**Keywords:** Epidémiologie, facteurs de risque, prévalence, troubles mentaux, pays en voie de développement, Epidemiology, risk factors, prevalence, mental disorders, developing countries

## Abstract

**Introduction:**

loin d´être un phénomène rare, les troubles mentaux représentent un enjeu majeur de santé publique. L´objectif de ce travail était d´étudier les caractéristiques épidémio-cliniques des patients consultants au service de psychiatrie de l´hôpital régional de Gabes, et d´identifier les facteurs de risque des principaux troubles mentaux.

**Méthodes:**

il s´agissait d´une étude rétrospective, ayant inclus tous les patients qui ont consulté pour la première fois au service de psychiatrie de Gabes entre le 01/01/2010 et le 31/12/2013. Les diagnostics ont été classés selon la 10ème version de la classification internationale des maladies de l´Organisation mondiale de la Santé (CIM10).

**Résultats:**

nous avons dénombré 1601 consultants. Le sexe ratio (H/F) était de 0,96. L´âge médian était de 34 ans (IIQ= [24-47,5 ans]). Le délai médian entre le début des symptômes et la consultation au service de psychiatrie était de 1 an (IIQ= [3mois-2 ans]). Les trois diagnostics les plus fréquemment posés étaient les troubles dépressifs avec une prévalence de 21%, suivis par la schizophrénie (10,6%) et le retard mental (9,7%). Les troubles dépressifs étaient significativement plus fréquents chez les consultants de genre féminin (p < 0,001), les consultants mariés (p < 0,001) et chez les adultes d´âge moyen ([40-65 ans]) (p < 0,001). La prévalence de ces troubles n´était pas significativement associée ni au niveau scolaire ni à l´activité professionnelle ni au niveau socio-économique.

**Conclusion:**

la connaissance du profil sociodémographique et clinique des consultants, ainsi que les troubles mentaux les plus fréquents et les facteurs qui leurs étaient associés permettrait de mieux adapter l´offre de soins à la demande et d´identifier les besoins en termes de formation en santé mentale au Sud-Est tunisien.

## Abstract (en)

**Introduction:**

mental disorders are a very common health condition and a major public health issue. The purpose of this study was to examine the epidemiological and clinical characteristics of patients coming for consultation to the psychiatric department of the Regional Hospital of Gabes, and to identify risk factors for the main mental disorders.

**Methods:**

we conducted a retrospective study, including all patients consulting the psychiatry department of Gabes for the first time between 01/01/2010 and 31/12/2013. Diagnoses were classified according to the 10^th^ reversion of the WHO International Classification of Diseases (ICD10).

**Results:**

one thousand one hundred and one consultation cases were included in this study. The sex ratio (M/F) was 0.96. The average age of patients was 34 years (IQR=24-47.5 years), the median time between symptom onset and consultation to the psychiatric department was 1 year (IQR=3months-2 years). The three most common diagnosed were depressive disorders (21%), followed by schizophrenia (10.6%) and mental retardation (9.7%). Depressive disorders were significantly more common among female patients (p<0.001), married patients (p<0.001) and middle-aged adults (40-65 years) (p<0.001). The prevalence of these disorders was not significantly associated with either educational level, professional activity or socio-economic level.

**Conclusion:**

the knowledge of the socio-demographic and clinical profile of the most frequent mental disorders and factors associated with them would make it possible to better adapt the supply of care to the demand and to identify the needs in terms of mental health training in South-East Tunisia.

## Introduction

Loin d´être un phénomène rare, les troubles mentaux représentent un enjeu majeur de santé publique. Selon l´Organisation mondiale de la Santé (OMS), environ 450 millions souffrent actuellement de perturbations mentales, allant des manifestations les plus légères aux pathologies les plus lourdes [1]. Elles sont universelles, touchant aussi bien les hommes que les femmes, à tous les stades de leurs vies, les riches comme les pauvres, en milieu urbain comme en zone rurale [1]. Des revues publiées en 2010 et en 2014 dans le cadre des enquêtes de l´OMS sur la santé mentale dans le monde (WMH Survey Initiative) ont présenté des chiffres alarmants [2,3]. Elles ont estimé qu´approximativement un individu sur 5 était atteint d´un trouble mental (la prévalence étant égale à 17,6%) et que presque 29,2% de ceux-ci étaient susceptibles d´être atteints par un tel trouble durant leur vie. A ces données s´ajoute le fait que 3 sur 4 malades mentaux atteints de pathologies psychiatriques graves ne bénéficiaient d´aucune prise en charge [4]. Outre le défi pour la santé publique, les troubles mentaux se présentent comme un fardeau pesant lourd sur les plans social et économique. Source de handicap majeur, les charges des maladies psychiatriques sont énormes. En effet, les troubles mentaux sont les premières causes des années vécues en inactivité (AVI) dans le monde et comptent pour 28,5% du total [5].

Toutefois, l´épidémiologie psychiatrique n´a pas évolué comme il se devait [6]. Cette discipline a eu une émergence tardive et son développement s´est heurté à divers obstacles. Un financement limité [7], une marginalisation par les programmes de santé au profit de la recherche biomédicale [8] et un chevauchement avec d´autres sciences comme la sociologie et l´anthropologie [9], telles étaient les difficultés rencontrées par cette branche d´épidémiologie. C´est à ce propos, que nous pouvons constater la pénurie, dans notre pays, des études épidémiologiques relatives aux troubles mentaux. Dans cette optique, les objectifs de notre étude étaient d´étudier les caractéristiques épidémio-cliniques des patients consultants au service de psychiatrie de l´hôpital régional de Gabes, et d´identifier les facteurs de risque des principaux troubles mentaux.

## Méthodes

**Type d´étude:** il s´agissait d´une étude rétrospective, réalisée au service de psychiatrie de Gabes entre le 1^er^ janvier 2010 et le 31 décembre 2013.

### Population d´étude

Cette étude s´est déroulée au service de Psychiatrie de l´Hôpital Régional de Gabès (au Sud Tunisien). Ce service assure la prise en charge externe des malades souffrant de troubles mentaux. L´unité d´hospitalisation n´a pas encore démarré au moment de l´étude. L´hôpital régional de Gabès est un hôpital général. La consultation de psychiatrie de cet hôpital est une consultation de deuxième ligne, le malade est adressé par le médecin de famille ou par un autre spécialiste ou intervenant, tel que le psychologue. Comme il s´agit du seul service spécialisé dans le Sud-Est de la Tunisie, les demandes émanent de plusieurs régions. L´équipe médicale était constituée de deux psychiatres hospitalo-universitaires et d´un médecin généraliste. Tous les patients qui ont consulté pour la première fois au service de psychiatrie de Gabes durant la période de l´étude ont été inclus. Nous avons exclu les patients dont le suivi, suite à la première consultation, n´a pas été indiqué (demande de certificat, expertise psychiatrique, etc.). Les patients qui avaient des dossiers médicaux inexploitables ont été aussi exclus de l´étude.

**Recueil de données:** les données ont été recueillies à l´aide d´une fiche préétablie après le consentement éclairé des patients de manière anonyme et confidentielle. Pour chaque patient, nous avons recueilli les données épidémiologiques (genre, âge, statut matrimonial, niveau scolaire, activité professionnelle, niveau socioéconomique) et cliniques (antécédents, conduites addictives, le motif de consultation, le diagnostic). Les diagnostics ont été classés selon la classification internationale des maladies (CIM 10) de l´OMS [10].

**Analyse statistique:** les données recueillies ont été saisies et analysées à l´aide du logiciel SPSS version 17.0. La description des données qualitatives était faite par le calcul de pourcentages. Pour les variables quantitatives, la normalité de la distribution était vérifiée par le test de Kolmogorov-Smirnov et le test de Shapiro-Wilk. Une estimation des moyennes avec leurs écarts types et de la médiane avec les intervalles inter-quartiles (IIQ) a été ainsi réalisée. Le test Chi-Deux de Pearson ou le test exact de Fisher (si les effectifs théoriques du tableau de contingence étaient < 5) étaient utilisés pour la comparaison des proportions. Le seuil de significativité a été fixé à 5%.

**Considérations éthiques:** le Comité de Protection des Personnes du Sud Tunisien (C.P.P.SUD) a approuvé le protocole de l´étude le 29 septembre 2021. La référence du comité est CPPSUD N° 0353/2021. L´étude suit les principes énoncés dans la déclaration d´Helsinki.

## Résultats

### Description de la population d´étude

Durant la période de l´étude, 1601 consultants ont été colligés, avec un sex-ratio à 0,96 et un âge médian de 34 ans (IIQ= [24 ans-47,5 ans]). La tranche d´âge des adultes jeunes ([18-40 ans]) était la plus représentée (n= 1289; 80,5%). Parmi les consultants, 789 sujets (51,7%) étaient célibataires et 638 (41,8%) étaient mariés. La majorité des consultants avait un niveau scolaire primaire (n=656; 45,1%), un niveau socioéconomique bas (n=1152; 77,9%) et était sans activité professionnelle (n=751; 49,3%). Quatre cents soixante-dix cas (29,3%) avaient des antécédents familiaux psychiatriques parmi lesquels le trouble bipolaire et le trouble dépressif étaient les plus fréquents avec respectivement 3,1% (n=49) et 2,6% (n=42). Les antécédents familiaux de suicide étaient notés chez 34 consultants (2,1%). Les antécédents médicaux étaient notés chez 399 consultants (24,9%) parmi lesquels 8,1% étaient hypertendus (n=129) et 6,2% étaient diabétiques (n=99). Le nombre total des consultants ayant un antécédent de suivi psychiatrique était de 413 (25,8%). La présence d´antécédents judicaires a été observée chez 59 patients (3,7%).

Parmi les consultants, 48 cas (3%) avaient des antécédents de tentative suicidaire (3%) et 288 cas (18%) ont eu recours à des conduites addictives. Le tabagisme était la conduite la plus fréquente (n= 270; 16,7%) suivi de la consommation d´alcool (n=48; 2,8%) et de psychotropes (n=8; 0,5%). Les motifs de consultation étaient le suivi d'une pathologie psychiatrique connue et équilibrée dans 18,5% des cas (n=296), suivi des troubles du comportement (n=201; 12,6%) et de l´anxiété (n=180; 11,2%). Cinquante-neuf patients (3,7%) ont été adressés suite à une tentative de suicide. Le délai médian entre le début des symptômes et la consultation au service de psychiatrie était de 1 an (IIQ = [3 mois-2 ans]) (Tableau 1). La répartition des troubles mentaux selon la CIM-10 a révélé que les troubles de l´humeur occupaient le premier rang (n=502; 31,4%) suivis par les troubles névrotiques (n=372; 23,2%), la schizophrénie, troubles schizotypiques et troubles délirants (n=269; 16,8%) et le retard mental (n=156; 9,7%). Les trois diagnostics les plus fréquemment posés étaient les troubles dépressifs avec une prévalence de 21%, suivis par la schizophrénie (10,6%) et le retard mental (9,7%) (Tableau 2).

**Tableau 1:** caractéristiques épidémiologiques et cliniques des nouveaux consultants au service de psychiatrie de Gabes

Variables	Nombre	Pourcentage
**Total**	**1601**	**100**
**Genre**		
Hommes	784	49
Femmes	817	51
**Classes d’âge (années)**		
< 18	147	9,2
[18-40]	824	51,5
[40-65]	505	31,5
≥ 65	125	7,8
**Statut matrimonial**		
Marié (e)	638	41,8
Célibataire	789	51,7
Divorcé (e)	52	3,4
Veuf (ve)	47	3,1
**Niveau scolaire**		
Analphabète	198	13,6
Primaire	656	45,1
Secondaire	396	27,3
Universitaire	293	14
**Niveau socioéconomique**		
Bas	1152	77,9
Moyen	320	21,7
Elevé	6	0,4
**Antécédents familiaux psychiatriques**	470	29,3
**Antécédents judiciaires**	59	3,7
**Conduites addictives**	288	18

**Tableau 2:** prévalence des troubles mentaux diagnostiqués dans la population d’étude

Diagnostic	Nombre de cas	Prévalence (%)
**Total**	**1601**	**100**
**Troubles de l’humeur**	**502**	**31,4**
Troubles dépressifs	336	20,9
Troubles bipolaires	152	9,5
Autres troubles de l'humeur	14	0,9
**Troubles névrotiques, troubles liés à des facteurs de stress et troubles somatoformes**	**372**	**23,2**
Troubles de l'adaptation	147	9,2
Troubles anxieux	145	9,05
Troubles somatoformes	34	2,1
Troubles dissociatifs	23	1,4
ESPT*	16	0,99
TOC*	7	0,4
**Schizophrénie, troubles schizotypiques et troubles délirants**	**269**	**16,8**
Schizophrénies	170	10,6
Troubles schizo-affectifs	38	2,4
Troubles délirants	21	1,3
Psychoses non organiques sans précision	40	2,5
**Retard mental**	**156**	**9,7**
**Diagnostics différés**	**102**	**6,4**
**Troubles mentaux organiques**	**56**	**3,5**
Démence	51	3,2
Autres troubles mentaux organiques	5	0,3
**Troubles de la personnalité et du comportement chez l'adulte**	**49**	**3,1**
Troubles de la personnalité	47	2,9
Pathomimie	2	0,1
**Troubles somatiques divers**	**34**	**2,1**
**Troubles mentaux diagnostiqués à l'enfance**	**26**	**1,6**
Enurésie	21	1,3
Encoprésie	4	0,2
Autisme	1	0,06
**Syndromes comportementaux associés à des perturbations physiologiques et à des facteurs physiques**	**21**	**1,3**
Insomnies anorganiques	14	0,9
Dysfonctionnements sexuels	6	0,4
Anorexie mentale	1	0,06
**Troubles mentaux et du comportement liés aux substances psycho-actives**	**14**	**0,9**
Alcool	6	0,4
Solvants volatils	4	0,2
Drogues multiples et autres substances psychoactives	4	0,2

ESPT: état de stress post-traumatique; TOC: troubles obsessionnels compulsifs

### Facteurs de risque des trois principaux troubles mentaux

Les troubles dépressifs étaient significativement plus fréquents chez les consultants de genre féminin (p < 0,001), les consultants mariés (p < 0,001) et chez les adultes d´âge moyen [40-65 ans] (p < 0,001). La prévalence de ces troubles n´était pas significativement associée ni au niveau scolaire ni à l´activité professionnelle ni au niveau socio-économique (Tableau 3). Nous avons noté une différence statistiquement significative de la prévalence de la schizophrénie selon le genre (p< 0,001), l´âge (p< 0,001), le statut matrimonial de non marié (p< 0,001), le niveau scolaire (p=0,037) ainsi que le niveau socio-économique bas (p< 0,001). Les consultants ayant des conduites addictives (p< 0,001) et ceux ayant des antécédents familiaux psychiatriques (p=0,02) étaient significativement plus à risque de développer la schizophrénie (Tableau 3).

**Tableau 3:** facteurs associés aux trois principaux troubles mentaux identifiés chez les consultants au service de psychiatrie de Gabes

Variables	Troubles dépressifs N (%)	Schizophrénie N (%)	Retard mental N (%)
**Genre**			
Hommes	120 (15,3)	113 (14,4)	94 (12)
Femmes	216 (26,4)	57 (7)	62 (7,6)
**p**	< 0,001*	< 0,001*	0,003*
**Classes d’âge (années)**			
< 18	17 (11,6)	4 (2,7)	31 (21,1)
[18-40]	137 (16,6)	114 (13,8)	101 (12,3)
[40-65]	150 (29,7)	49 (9,7)	23 (4,6)
≥ 65	32 (25,6)	3 (2,4)	1 (0,8)
**p**	< 0,001*	< 0,001*	< 0,001*
**Statut matrimonial**			
Marié(e)	199 (31,2)	44 (6,9)	7 (1,1)
Non marié(e)†	128 (14,4)	116 (13,1)	36 (15,3)
**p**	< 0,001*	< 0,001*	< 0,001*
**Niveau scolaire**			
Analphabète	51 (25,8)	10 (5,1)	40 (20,2)
Primaire	123 (18,8)	81 (12,3)	92 (14)
Secondaire	91 (23)	42 (10,6)	4 (1)
Universitaire	48 (23,6)	22 (10,8)	0
**p**	0,1	0,037*	< 0,001*
**Activité professionnelle**			
Absente	211 (20,7)	113 (11,1)	128 (12,6)
Présente	125 (21,4)	57 (9,8)	28 (4,8)
**p**	0,7	0,4	< 0,001*
**Niveau socio-économique**			
Bas	242 (21)	136 (11,8)	130 (11,3)
Moyen/Elevé	81 (24,8)	14 (4,3)	3 (0,9)
**p**	0,1	< 0,001*	< 0,001*
**Antécédents familiaux psychiatriques**			
Oui	100 (21,3)	63 (13,4)	45 (9,6)
Non	236 (20,9)	107 (9,5)	111 (9,8)
**p**	0,8	0,02*	0,9
**Antécédents judiciaires**			
Oui	7 (11,7)	4 (6,8)	1 (1,7)
Non	329 (21,3)	166 (10,8)	155 (10,1)
**p**	0,08	0,3	0,03*
**Conduites addictives**			
Oui	53 (18,4)	52 (18,1)	9 (3,1)
Non	283 (21,6)	118 (9)	147 (11,2)
**p**	0,2	< 0,001*	< 0,001*

N= nombre; %: pourcentage; *: Association statistiquement significative; †: célibataire, divorcé (e) et veufs (ve)

La prévalence du retard mental était significativement plus élevée chez les consultants de genre masculin (p=0,003), les enfants âgés de moins de 18 ans (p< 0,001), les analphabètes (p< 0,001), les sujets sans activité professionnelle (p< 0,001), et ceux ayant un niveau socio-économique bas (p< 0,001). Cependant, la prévalence du retard mental était significativement moins fréquente chez les sujets mariés (p< 0,001) (Tableau 3).

## Discussion

Dans notre population d´étude, la tranche d´âge des adultes jeunes était la plus représentée (51,5%), ce qui était concordant avec les données de la littérature [11,12]. Bien que seulement 12% des adultes jeunes déclarent avoir une maladie ou une invalidité de longue durée, les problèmes de santé mentale demeurent répandus chez cette tranche d´âge [12]. Il a été estimé que 75% des troubles mentaux émergent à l´âge du jeune adulte, souvent avant 25 ans [13]. En effet, la période de transition à la vie adulte, soit la période comprise entre 18 et 30 ans, est une période charnière marquée par l´acquisition de nouveaux rôles et responsabilités [14]. Le jeune adulte est alors confronté à de nombreux changements sur les plans biologique, académique, professionnel et psychologique. Il s´agit d´une période critique fréquemment à l´origine d´une détresse psychologique pouvant affecter la santé mentale [15]. Il serait ainsi nécessaire d´assurer une prise en charge spécifique à cette tranche d´âge mettant le focus sur la facilité d´accès aux soins, l´adaptation aux problématiques du jeune adulte, avec approches médicale et psychosociale intégrées, afin de diminuer le risque d´évolution vers une pathologie chronique et améliorer le fonctionnement global de l´individu [16].

Bien que les troubles mentaux émergent fréquemment à l'âge du jeune adulte, une prise en charge n'est généralement entreprise que quelques années plus tard, à un stade où il est souvent difficile de pouvoir restaurer un équilibre satisfaisant [17]. Dans notre étude, le délai médian entre le début des symptômes et la consultation au service de psychiatrie était de 1 an. Ce délai était largement inférieur à celui trouvé dans une étude menée aux Etats Unis où 80% des personnes atteintes d´un trouble mental finissaient par avoir un traitement adéquat après un délai moyen de plus de dix ans [18]. D´autres études antérieures ont documenté une longue durée de psychose non traitée, d´environ deux ans [19] pour la schizophrénie, de 1à 14 ans pour les troubles de l´humeur et allant jusqu´à 30 ans pour les troubles anxieux [20]. Ces études ont également mis en évidence que plus long est le délai entre l´apparition de la maladie et l´instauration d´un traitement efficace, moins bon est le pronostic. Il faudrait ainsi intervenir dans la phase précoce des troubles mentaux pour améliorer les soins auprès d´une population de jeunes patients à risque d´évolution chronique en cas de traitement inadéquat.

Notre étude a montré que les trois diagnostics les plus fréquents étaient par ordre décroissant les troubles dépressifs suivis par la schizophrénie et le retard mental. Toutefois, tant aux États-Unis qu´en Europe, les diagnostics les plus courants dans les établissements psychiatriques étaient les troubles de l´humeur, les troubles anxieux et les troubles somatoformes [20]. L´importance des troubles mentaux en termes de fréquence et l´ordre étaient parfois différents entre les pays. En effet, la répartition respective de ces trois diagnostics était de 9,5%,18,1% et 8,9% aux États-Unis, 13,5%, 16,4% et 35,9% au Danemark [21], 31%,19% et 18% en Belgique [22] et de 16,5%, 13,5% et 11,3% en France [23]. Par ailleurs, en Afrique du Sud et au Nigéria, les troubles mentaux et du comportement liés aux substances psychoactives occupaient le 2^e^ rang après les troubles anxieux avec des prévalences respectives de 13,3% [24] et 3,9% [25]. Cette variabilité de la prévalence des troubles mentaux entre les pays serait probablement influencée par les différences méthodologiques d´une part et les populations étudiées d´autre part. En effet, notre étude s´est déroulée dans un service de psychiatrie dans un hôpital général. En plus, vu l´absence d´un pédopsychiatre, plusieurs troubles mentaux de l´enfant sont pris en charge dans le même service. Cela pourrait expliquer la prévalence élevée du retard mental parmi les diagnostics.

La prédominance féminine dans la dépression était l´une des notions les plus prouvées en épidémiologie psychiatrique, avec une prévalence pouvant atteindre jusqu´à trois fois celle des hommes [26]. Dans notre étude, la prévalence des troubles dépressifs était significativement plus élevée chez les sujets de genre féminin, ce qui était en concordance avec les données de la littérature [22,27]. Une étude hongroise a expliqué la prédominance féminine par une comorbidité entre troubles dépressifs et anxieux, particulièrement importante chez les femmes [28].

Dans la présente étude, le statut matrimonial de marié était significativement associé aux troubles dépressifs. En revanche, d´autres études ont montré que le statut de non marié, sans distinction entre veufs et divorcés ou célibataires, était associé à la survenue de ces troubles [29,30]. Par ailleurs, de rares études n´ont pas mis en évidence une différence significative de la prévalence des troubles dépressifs selon le statut matrimonial [31]. Dans notre étude, la prévalence des troubles dépressifs n´était pas significativement associée au niveau scolaire des participants. Ce résultat a été aussi trouvé dans une étude menée en Europe [32]. Toutefois, dans une étude brésilienne la prévalence des troubles dépressifs était trois fois plus basse chez les sujets ayant un haut niveau scolaire [30].

La majorité des études s´est intéressée à étudier l´association entre l´activité professionnelle et la prévalence des troubles dépressifs. Or, contrairement à notre étude où cette association n´était pas statistiquement significative, des études antérieures ont établi l´implication de l´absence d´activité professionnelle dans le risque de survenue des troubles dépressifs [33,34].

En règle générale, les conduites addictives sont plus fréquentes en populations psychiatriques qu´en population générale [35]. Dans le même ordre d´idées, nous avons noté une association statistiquement significative entre la schizophrénie et les conduites addictives. Selon des études antérieures les substances les plus fréquemment utilisées étaient le cannabis, l´alcool et le tabac [36]. Des études longitudinales récentes, ont clairement établi que le cannabis était facteur de risque de schizophrénie avec un effet dose-dépendant [37]. Le tabagisme est également un problème très important de santé publique chez les patients souffrant de schizophrénie. Une méta-analyse a conclu que la consommation quotidienne de cigarettes était associée à un risque accru de psychoses, dont la schizophrénie, mais aussi à l'apparition plus précoce de ces troubles [38]. L´alcool, une autre substance psychoactive, a été signalé comme étant abusé par 50% des patients atteints de schizophrénie [39]. La présence de conduites addictives chez les patients schizophrènes complique leur prise en charge et diminue leur qualité de vie [35]. Une prise de conscience est ainsi nécessaire chez les psychiatres, qui sous-estiment l´impact des conduites addictives chez leurs patients schizophrènes, et chez les soignants des centres de soins spécialisés en toxicomanies, qui diagnostiquent difficilement des troubles schizophréniques chez les sujets toxicomanes.

## Conclusion

Les troubles mentaux représentent un enjeu majeur de santé publique et se présentent, en plus, comme un fardeau pesant lourd sur les plans social et économique. La connaissance du profil sociodémographique et clinique des consultants, permettrait de mieux adapter l´offre de soins à la demande et d´identifier les besoins en termes de formation en santé mentale au sud-est tunisien. La connaissance des troubles mentaux les plus fréquents dans notre population d´étude ainsi que les facteurs qui leurs étaient associés constitue une étape cruciale pour la mise en place des mesures de prévention efficaces et améliorer le pronostic et la qualité de vie des patients.

### Etat des connaissances sur le sujet

- *La majorité des troubles mentaux apparait à l´âge du jeune adulte, période critique fréquemment à l´origine d´une détresse psychologique pouvant affecter la santé mentale;*
- *Bien que les troubles mentaux émergent fréquemment à un âge jeune, une prise en charge n'est généralement entreprise que quelques années plus tard, à un stade où il est souvent difficile de pouvoir restaurer un équilibre satisfaisant*.

### Contribution de notre étude à la connaissance

- *Les trois diagnostics les plus fréquemment posés étaient les troubles dépressifs qui étaient significativement plus fréquents chez les consultants de genre féminin, les consultants mariés et chez les adultes d´âge moyen [40-65 ans], suivis par la schizophrénie et le retard mental;*
- *Le délai médian entre le début des symptômes et la consultation au service de psychiatrie était de 1 an;*
- *Toutes ces données sont importantes à considérer afin d´orienter des travaux de dépistage chez les populations à risque; cela permettra de raccourcir le délai entre le début des symptômes et la consultation*.
